# “*Hitting the spot*”: Developing individuals with lived-experience of health and social care as facilitators to deliver a course to enhance public involvement in research – a Welsh perspective

**DOI:** 10.1186/s40900-017-0057-z

**Published:** 2017-04-04

**Authors:** Alan Meudell, Sian Jones, Natalie Simon, Zoe Hunter, Barbara Moore, Jim Elliott, Dawn Casey

**Affiliations:** 1 Involving People Network, Health and Care Research Wales, Cardiff, UK; 2 Health and Care Research Wales, Cardiff, UK; 3 HRA, London, UK; 4 Macmillan Cancer Support, London, UK

**Keywords:** Patient and public involvement, Public involvement in research, Public involvement training, Wales, Involving people network, Building research partnerships

## Abstract

**Plain English summary:**

Public involvement in research has become an important and integral part of the research process in health and social care, from the early stages of research prioritisation and development to the later stages of research conduct and dissemination.

Learning and development opportunities, including training, can assist the public and researchers in working together in the research process, and a training schedule exists in Wales for this purpose. One of the key components of this training schedule in Wales is the course *Involving the Public in the Design and Conduct of Research: Building Research Partnerships.*

Building on the existing successes of this UK-wide course, first developed by Macmillan Cancer Support, a project was established between Health and Care Research Wales and Macmillan Cancer Support to develop three members of the Involving People Network into trained facilitators. Once trained, the aim was for the three facilitators to deliver the course in Wales.

Macmillan Cancer Support and Health and Care Research Wales selected, through a competitive process, three members of the Involving People Network to use their lived experience of Involvement in research projects, as well as any lived experience of a physical or mental health condition or illness, to become facilitators of the course in the unique context of public involvement in research in Wales.

Through this process many benefits were realised, including developing the course content and its delivery in Wales, as well as building the skills and confidence of the individuals themselves as facilitators. This has contributed to a continuing commitment to the sustainable delivery of the *Involving the Public in the Design and Conduct of Research: Building Research Partnerships* course in Wales and a combined approach to addressing any challenges and obstacles which presented.

**Abstract:**

Health and Care Research Wales has a strategic aim to *Ensure public involvement and engagement is central to what we do and visible in all elements of it.* As part of the ongoing development of the Health and Care Research Wales Training Programme a project was initiated to develop members of the public as facilitators to deliver a public involvement in research course.

The project was undertaken in collaboration with Macmillan Cancer Support and was advertised via the Involving People Network in Wales. Three trainee facilitators were recruited, from 14 people that applied, to deliver a public involvement in research training course, the *Building Research Partnerships* course, as it was known then, originally developed for and by Macmillan Cancer Support.

As members of the Involving People Network, the trainees were given training, mentorship, financial and administrative support to develop their role as facilitators over a two year period. This has been reciprocated with incredible commitment, ongoing course delivery in Wales, excellent course evaluations, course review and involvement in future planning.

Through this project several benefits were realised, including developing the course content and its delivery and building the skills and confidence of the individual facilitators themselves. Additionally, and importantly, the project team found that patients and members of the public who are given appropriate training and support can greatly enhance a research training programme and act as highly effective ambassadors to further the cause of public involvement in research.

**Electronic supplementary material:**

The online version of this article (doi:10.1186/s40900-017-0057-z) contains supplementary material, which is available to authorized users.

## Background

Health and Care Research Wales has a strategic aim to *Ensure public involvement and engagement is central to what we do and visible in all elements of it* [1]*.* As part of the ongoing development of the Health and Care Research Wales Training Programme [2] a project was initiated to develop members of the public as facilitators to deliver a public involvement in research course.

The project was undertaken in collaboration with Macmillan Cancer Support [3] and was advertised via the Involving People Network [4] in Wales. Three trainee facilitators were recruited to deliver a public involvement in research training course, the *Building Research Partnerships* course [5], as it was known then, originally developed for and by Macmillan Cancer Support.

## Public involvement in health and social care research

The importance of involving the public in research is well-recognised and advocated in the Research Governance Frameworks for England [6] and Wales [7]. Whilst some professional and organisational barriers to public involvement do exist [8], there also exists strategic infrastructure support for public involvement in research. This includes the establishment of the Health and Care Research Wales Public Involvement and Engagement function [9] in Wales, hosting the Involving People Network [4], and the National Institute for Health Research (NIHR) funding for INVOLVE [10] in England.

There can be a range of positive outcomes of public involvement in research, including identification of research questions and improved study recruitment, amongst many others [11]. A recent review of public involvement in the National Institute for Health, Going the Extra Mile [12], has celebrated the value and achievements of public involvement in NIHR funded research and has set an agenda for moving forward.

## The landscape of public involvement in research in Wales

Health and Care Research Wales strives for an environment in which all health and social care research that takes place in Wales happens with the public and for the public [1]. Central support for this vision is provided by the Public Involvement and Engagement function providing high quality guidance, resources and training for researchers, the public, and health and social care professionals across Wales.

Involvement and engagement of the public in research in Wales is widespread, for example a paper published in the British Journal of Psychiatry in 2014 [13], reports that 23 service users were involved in mental health research in Wales.

The primary mechanism for public involvement in research in Wales is through the Involving People Network, and membership includes individuals with a range of interest areas from cancer, mental health and diabetes, to ageing and emergency care. In 2015-2016 eighty-three research involvement opportunity places were filled by Involving People Network members. Individuals on the Network can access a range of opportunities for involvement, including reviewing research grant applications to Welsh Government and charities, becoming members of research development groups, assistance with data collection, for example as service user research assistants. These involvement activities are supported by Health and Care Research Wales through training, communication, specialist advice and guidance, reimbursement of expenses and payment for time.

## Training and support for public involvement in research

We acknowledge that the term ‘training’ can be used to describe a wide range of activities which help people develop skills, experience and knowledge, including attending conferences, participating in workshops, undertaking online activities. In this paper we use the term ‘course’, when referring to *Involving the Public in the Design and Conduct of Research: Building Research Partnerships*.

The course, which has evolved over time from the original Macmillan Cancer Support Building Research Partnerships [5] (the original manual ID dated 2007), has been delivered in Wales over a number of years. Initially entitled in Wales as *Getting Involved and Influencing Research,* the course has evolved from a cancer-focused course into a course adapted for Involving People Network members and researchers working across a wide range of health conditions. The current course is a one-day training course, available to individuals including researchers, health professionals, policy makers, and Involving People Network members, via the Health and Care Research Wales training programme. The aim of the course is to introduce the concepts and practice of involving the public in research. Involving People Network members are encouraged to attend to fulfil their membership and adhere to the Involving People Network code of conduct.

Attendance on the course is free for researchers working in health and social care research in Wales, and free also for members of the Involving People Network, with the latter group receiving additional support for their attendance by having their travel and subsistence needs booked or reimbursed to enable them to attend training.

## Value of the lived experience

From the outset of the development of the Macmillan course, public facilitators have brought their lived experience to the role and the courses they facilitate [5]. The project described in this paper was initiated because this was the first time Involving People Network members had been invited to facilitate the course. It was felt that Network membership was a necessary requirement to the effective facilitation of the course in Wales, given the understanding Network members would have about the landscape of public involvement in research in the unique, devolved Welsh health and social research infrastructure. The project also offered an innovative approach to the development and delivery of training courses which in turn would help build capacity for course facilitation across Wales.

An important potential benefit of the project was building the skills and confidence of the individuals themselves, through their involvement and development as facilitators. Similarly, by having a mix of researchers and public/patients on each course, it gave the delegates an opportunity to exchange views on involvement in research and gain an understanding of each other’s viewpoint.

Research suggests that in addition to the reported benefits of public involvement to the research process itself, there are benefits to individuals themselves when getting involved. For example a 2002 paper, by Rhodes et al [14], showed that service users involved in a research advisory group reported feeling greater confidence when involved.

## The project

The project aimed to recruit and train three Involving People Network members to deliver the training course *Involving the Public in the Design and Conduct of Research: Building Research Partnerships*, in Wales. The project team’s objectives were to support three Involving People Network members to co-facilitate the course in Wales, as well as involving the facilitators in the review and development of the course, to inform future plans ensuring ongoing development of the facilitators and long-term sustainability of the course in Wales.

A memorandum of understanding was developed in July 2013 between Macmillan Cancer Support and Health and Care Research Wales (formerly National Institute for Social Care and Health Research Clinical Research Centre), to clarify roles and expectations. In September 2013 an advert was developed which was shared with the Involving People Network in Wales. Through a competitive selection process, Health and Care Research Wales and Macmillan Cancer Support shortlisted from the 14 applications received and subsequently, three network members were selected for the role. The main selection criteria for the role were:

- Experience of involvement in research projects.
- Personal lived-experience of a physical or mental health condition or illness.
- Facilitation, communications and people skills

Between November 2013 and November 2015 the selected facilitators received training, support and opportunities to co-facilitate in order to develop in their roles. A Lead Facilitator, who was already facilitating courses in England, offered ongoing support and feedback and co-facilitated courses with each facilitator. Each facilitated course was evaluated in a consistent way [see Additional file 1].

Health and Care Research Wales provided the support function of the project, this included financial support for the expenses of the facilitators, taking on the administration and costs of course delivery, including advertising courses, registering delegates, preparing course materials, booking and providing the venue and catering for each course. This was in line with a Memorandum of Understanding document developed at the outset of the project. In December 2015 the final project review meeting took place, and an action plan detailing the next steps for continued support for the facilitators was developed and agreed.

Although the project has taken longer than originally envisaged, the project has achieved its aims. There are now three trained Involving People Network facilitators who have been empowered to develop the course, its materials and its title. For example the facilitators incorporate their own examples of recent public involvement practice to demonstrate impact. As of January 2015 the course delivered in Wales has been entitled *Involving the Public in the Design and Conduct of Research: Building Research Partnerships,* this name change decision was agreed in order to better communicate the intended purpose of the course.

The one day course comprises of a number of sessions as can be seen in Table 1. Whilst the course structure does not alter, there is an ability for content of some of the sessions to be changed to make it more relevant to the delegates attending the course. This is done at the discretion of the facilitators delivering the course on that day.

**Table 1:** Course structure

am	pm
Registration	Group work: case examples.
Welcome, introductions and expectations	Working with others
What is research?	Finding people to involve in research
Introduction to research language	Pass it on – action planning and evaluation.
What is public involvement and why is it important?.	Close
The research cycle and public involvement	

The course delegate pack has been developed and seven courses have been delivered in Wales between November 2013 and November 2015. At the present time the authors believe that the majority of public involvement training courses run across the UK are not run solely by members of the public, whereas the *Involving the Public in the Design and Conduct of Research: Building Research Partnerships* courses in Wales are.

Through liaison with NIHR in England the authors have ensured the courses run in Wales are promoted on the NIHR website [15]. Information on the project has been shared publicly including a presentation at the 2014 INVOLVE conference [16], and a model for facilitator development was developed and shared at a presentation at the 2016 NHS England Annual Research & Development conference [17]. The stages of the facilitator model are outlined in Table 2 but the full model can be seen as an additional file [see Additional file 2].

**Table 2:** Facilitator model

Observation and mentorship	
Co-facilitation with lead facilitator	
Progress Review	
Lead facilitator observation of facilitation	
Full facilitation	

The seven courses which were delivered in Wales during the project phase were evaluated. Six of these courses were evaluated using the standard Health and Care Research Wales evaluation form [see Additional file 1] and 92% of delegates at these sessions evaluated the course as good or excellent as shown below in Table 3:

**Table 3:** Evaluation summaries

% Evaluations								
Date	Number of delegates	Number of Involving People Network members	Number of researchers	Poor	Satisfactory	Good	Excellent	Good or Excellent
14.11.13	14	11	3	0%	8%	25%	67%	92%
27.3.15	11	3	8	0%	18%	37%	45%	82%
18.6.14	9	2	7	0%	0%	33%	67%	100%
28.8.14^a^	7	3	4					
17.3.15	13	6	7	0%	10%	20%	70%	90%
9.7.15	19	10	9	0%	5%	26%	68%	95%
13.11.15	12	4	8	0%	8%	42%	50%	92%
TOTALS	85	39	46	0%	8%	30%	62%	92%

^a^this course ran for facilitator development, delivered to a small invited audience. Detailed feedback was given to the facilitators in discussion with their mentor, therefore the session was not evaluated or recorded in the usual way

In addition the facilitators were encouraged to complete a self-evaluation form [see Additional file 3], after each course for their own reflective learning.

## Discussion

All three facilitators have demonstrated ongoing enthusiasm and commitment to the training and the course and their development as facilitators. This has been made possible by the ongoing support and feedback of the lead facilitator who in turn has been supported in this role by Macmillan Cancer Support, and through support from Health and Care Research Wales who have supported the expenses of the facilitators and course administration and delivery costs.

The course is flexible, participatory and can be tailored to different groups. The course can work with a variety of group size and mix of people as long as there is a solid representation from Involving People Network members.

The facilitators have been empowered to develop the course, its materials and the title of the course. This has been one of the more positive aspects of the process in that the three facilitators have been able to draw on their own experiences of their involvement in the research process to develop the course content. It is that familiarity with, and ownership of, the course content that they feel has contributed to the levels of positive feedback from the delegates, as evidenced in the delegate feedback (see the Evaluation and Perspectives section) Also, anecdotally, the facilitators feel that this has had a positive impact on their confidence in their ability to deliver the course. It has brought about a growing confidence in the course material, particularly when they have been reviewed within the project team as a whole.

Drawing on their own experiences of involvement in research, the facilitators developed course materials such as scenarios that demonstrate what can happen between researchers and lay people when working together in a research project through to in-depth case studies of research projects where public and patient involvement has worked.

The flexibility of the course enables the facilitators that are delivering the course to adapt the sessions so that they can “play to their strengths” in certain sessions.

However, as with any project of this nature, there were challenges in certain areas. Whilst they were all resolved, there were a number of key learning points which were acknowledged and, they in turn, influenced the recommendations that can be found in that section.

### Following recruitment

Following notification of success of their application to become facilitators, the facilitators would have valued a meeting to meet each other, to talk through the training process, discuss what was expected of them and answer any questions they may have. This should have been augmented by a facilitator information document for each facilitator.

### Ongoing course delivery

A single point of contact for communication was agreed as it was not originally clear to the facilitators where responsibilities lay. After, discussion between the project team and the facilitators, this was agreed as being Health and Care Research Wales.

During the facilitator training period of the project, face-to-face meetings before each course were instigated with all facilitators to agree contributions, who does what etc. These replaced telephone meetings. However, it is interesting to note that whilst the facilitators valued the face-to-face meetings during the training period of the project, they have developed such a good working relationship with each other that, if necessary, they are happy to hold the pre-course meetings with each other by telephone.

Ongoing communication after each course has been seen as essential. This includes feedback from the co-facilitators, opportunity for group discussion amongst the facilitators concerning any new course material that has been used or any learning points that may have arisen during the course. It also includes feedback on the delegate evaluations and next steps. The facilitators have needed a continuing facility for production of existing hand-outs, resources, laminates as well as any resources needed for new course material developed by them etc. This is being provided by Health and Care Research Wales.

### Frequency of courses

Timeliness of the courses is crucial for continuing skill development of the facilitators. Efforts need to be made to ensure there is not too long a gap between courses. It has been a continuing challenge in Wales to deliver courses in a timely manner, as demand has at times been low. Two courses were cancelled, meaning there was a six-month gap in course delivery.

Changes in course title, marketing and the research infrastructure in Wales seems to have had a positive effect on demand for the course, but this needs further monitoring.

### Delegates

As mentioned previously, it can be difficult to achieve the desired mix of researchers and Involving People Network members and this will continue to be monitored. In principle, other members of the public attending via Macmillan Cancer Support advertising can access the training. However, Health and Care Research Wales funding (expenses and honoraria) is restricted to members of the Involving People Network.

Also, as part of the day, the course allows researchers to bring in documentation from their own research study, such as patient information sheets to be reviewed. However, they do not always respond to such requests which can present challenges on the day of training. However, the facilitators and Health and Care Research Wales have sourced a number of such documents that can be used when necessary, although any such documentation which is brought in by researchers on the day takes priority.

### Evaluation and perspectives

The facilitators were encouraged to complete self-evaluation forms for reflective learning [see Additional file 3] and we also collected their personal perspectives.

#### SJ’s perspectives

*“Since 2010 I have been an active Involving People Network member, committed to several trials/studies. When I completed the application form to become a facilitator I was overwhelmed to be chosen to become part of a team for the Involving the Public in the Design and Conduct of Research: Building Research Partnerships course. I was a little apprehensive and worried that I wouldn’t be able to fulfil the requirements expected of me.*

*Also at that time I was going through an emotive period. Thankfully the Training Team and the Public Involvement and Engagement Team didn’t give up on me as they supported me throughout.*

*The new course is really hitting the spot. It has been modified and is really delivering the message to the researchers, professionals, and Involving People members who are attending the course with really positive feedback. I have grown into the role as in the last three years my involvement as a lay representative has evolved hugely. I have learnt so much from various studies, trials, focus and discussion groups that I am involved with, I feel I can transfer some of my skills and experience into facilitating the course. I learn something new after every session, but one thing I do feel is important during the training is ‘Research Cycle’. I always find it interesting that many participants don’t understand where Public Patient Involvement fits: it’s always interesting to hear their surprise of the outcome. Another surprise is Participation, Involvement and Engagement, many don’t understand the differences, so that session is so informative.*

*We all work well as a team, supporting each other during the sessions; I feel confident that the course will now go forward in a positive way meeting the ever increasing needs of involvement in Health and Social Care Research in Wales”.*

#### AM’s perspectives

*“I have been an Involving People network member since 2008 and have benefitted from the opportunities that being a member has brought me, both from access to training through to working on several research projects in a number of roles.*

*When the opportunity to become a facilitator on the “Involving the Public in the Design and Conduct of Research: Building Research Partnerships” course was advertised, I applied and was successful. I felt it was an opportunity to pass on the various skills and knowledge I had gained in a number of roles within the field of research.*

*I liked the ‘hands on’ approach to training as it gave the opportunity to learn through co-facilitating the course alongside one of the experienced facilitators, progressively delivering more of the course as time went on. The other thing I like about the course is the fact that the way it is structured and the amount of course material we have access to allows us, as facilitators, a bit of flexibility when delivering it on the day. This enables us to tailor the course to better meet the needs of the delegates. The unexpected thing for me is the fact that although I am there as a facilitator, I have added to my knowledge of research from the interaction with the delegates”.*

#### JE’s perspectives

*“My role as lead facilitator was to guide, support and encourage the three new facilitators rather than to teach them as such. This is in keeping with one of the key principles of the original Building Research Partnerships course. As one of the people who developed the original course said, “The knowledge is in the room and your role is to help bring it out”.*

*Sian, Jacqui and Alan gained knowledge about the content and confidence in facilitating participants through the various exercises by first co-facilitating them with me and then doing them on their own, one by one. There is flexibility in the design of the course that allowed them to choose which exercises to facilitate and this was also important in building their confidence. A key point in their journey as facilitators for me was when they asked whether they could change some of the exercises and develop a new one. This showed me that they had fully understood what their role was as facilitators and how the materials available for the course could be used to help them.*

*Another feature of the course that was helpful in building their confidence was the use of case studies to illustrate the different ways patients and the public can be involved in research. It allowed them to contribute with examples from their own involvement in research. These were well received by delegates, as confirmed by the feedback, and that helped immeasurably in demonstrating to them that they were enabling delegates to learn about public involvement.*

*In preparing for each course we met to discuss and agree which parts each would facilitate. We also met briefly after each to review how they felt they had performed and give me the opportunity to provide feedback. This included suggestions for different ways they might facilitate the exercises. We developed a good rapport as we worked together and all along it felt like working with a team. It has been enjoyable and rewarding to see them all develop their skills and confidence as facilitators and contribute their own ideas to shape the course”.*

Data from completed evaluation forms of delegates is shown above in Table 3. However, the evaluation forms [see Additional file 1] also asked delegates to comment and reflect, and some of their perspectives are included below:

*“Fantastic group – thanks to having a good mix of research nurses, Involving People volunteers, Service users, research administrators etc – good mix”.*

*“Invaluable to a current project I am involved with”*

*“Far exceeded my expectations”*

*“Very interesting, good mix of individuals attending from a range of backgrounds. Enjoyed the group work sessions during the day. Good opportunities for interaction. Interesting discussions”*

*“Much more aware of what public involvement is…”*

*“Has given me some good checklists to help involve the public and how best to involve public and meet some potential public to get involved”*

*“Trainee facilitators were very good, would never have known they were trainees, they seemed like experts, well done!”*

*“Defined my role as lay representative”*

However, there were also comments that were not as positive:

*“A lot in one day, maybe 1.5 days and some activities built in”.*

*“I feel the day was too health focused”.*

*“It was great to be mixed for the day, but I think addressing specific issues in separate groups would have been beneficial”.*

*“I would have liked to hear more examples of how PI [Public Involvement] has been used and improved research for a bit of inspiration and ideas”*

From the personal perspectives and comments drawn from the reflections of the trainee facilitators and the lead facilitator, it can be seen that they have benefitted from their involvement in the project. Apart from the facilitators developing new skills, they have gained the confidence to deliver the course,﻿ as SJ said, ﻿'*I have grown i﻿nto the role'.*﻿﻿﻿The comments on the course structure and content are also positive, with the design of the course to be flexible being of particular note. The fact that the facilitators are able to make decisions on which course materials to use on the day to meet the needs of the delegates can be said to be empowering. This can also be said of the facilitators being encouraged to develop new course materials and content.

The evaluation of the course from the delegate feedback forms has also been positive with a high percentage of satisfaction being demonstrated. The comments from the delegates for the most part have been positive and where they have been negative, these were discussed in the post course meetings and used to inform next steps.

However, there is little evidence, as yet, of the long term benefit or impact that using members of the public with lived experience to facilitate the course has had on delegates receiving training and, in turn, public and patient involvement in research in Wales. This is due to three main factors.

Firstly, it is felt that only courses delivered by the facilitators post qualification can be used as evidence of impact. This is to factor out any influence, perceived or real, from the lead facilitator being present on the course. At this point it would limit the evidence to courses delivered in 2016 only.

Secondly, the delegates who were researchers were at varying places on their career paths and in varying roles, for example, a number of delegates were in mid – project or not as yet in a role where they could implement public and patient involvement in a research project.

Thirdly, the delegates who were members of the public, patients or their carers, are reliant on opportunities for involvement being generated by the research community. This, along with need to match the lived experience of the individual with a suitable research project, means that it may take time for suitable opportunities where delegates can use the knowledge gained from attending the course.

However, it is hoped that the benefits and impact that the approach of using members of the public to facilitate this course, as well as the course itself, will be examined as a research study once sufficient time has been allowed to start to mitigate the factors listed above.

## Recommendations

The project team developed recommendations, which Health and Care Research Wales and Macmillan Cancer Support will endeavour to follow in order to continue supporting the facilitators to deliver the course in Wales.

1. Health and Care Research Wales will continue to support and fund the facilitators in their development and to attend opportunities to share practice across the UK.
2. A facilitator agreement will be developed to outline facilitator, Health and Care Research Wales and Macmillan Cancer Support roles and responsibilities in terms of ongoing course development and delivery. This will include payment and support of the facilitators as part of the wider network of Health and Care Research Wales trainers.
3. Quarterly scheduling of courses across Wales should be instigated for 2016.
4. There should be annual review of the *Involving the Public in the Design and Conduct of Research: Building Research Partnerships* programme and associated materials.
5. Opportunities to collaborate across the UK in course development, evaluation and impact should be sought.
6. A mechanism for continuing feedback to Macmillan Cancer Support should be developed.

## Conclusion

The collaboration between Macmillan Cancer Support and Health and Care Research Wales has been extremely positive. There has been a continuing commitment to steer the project towards achievement of the sustainable delivery of the *Involving the Public in the Design and Conduct of Research: Building Research Partnerships* course in the context of the Health and Care Research Wales funded infrastructure. The collaboration has demonstrated the feasibility of training patients, carers and service users to facilitate the course, an approach that has been well received by delegates attending.

The recommendations offered and implemented will ensure the *Involving the Public in the Design and Conduct of Research: Building Research Partnerships* course remains a key component of the Health and Care Research Wales Training Programme.

The combined empowering and reflective approach to addressing challenges and obstacles has been realised. This has benefited the progress of the project and its outcomes. The Involving People Network trained facilitators have brought their lived experiences to the development of the course content and to the delivery of the course. Delegates who have attended the course have evaluated it very well and there have been benefits to the individual facilitators themselves through their development throughout the project.

## Additional files

Additional file 1: Evaluation Form (DOC 251 kb)

Additional file 2: Model of facilitator development (DOCX 13 kb)

Additional file 3: Self-evaluation form (DOCX 14 kb)

## Abbreviation

NIHR: National Institute for Health Research
