# Supplementary material for: “Hitting the spot”: Developing individuals with lived-experience of health and social care as facilitators to deliver a course to enhance public involvement in research – a Welsh perspective
Source: Res Involv Engagem. 2017 Apr 4;3:5. doi: 10.1186/s40900-017-0057-z (PMC5611615; doi:10.1186/s40900-017-0057-z)
Supplement: Supplementary file 1 — Evaluation Form (DOC 251 kb) [file 40900_2017_57_MOESM1_ESM.doc]

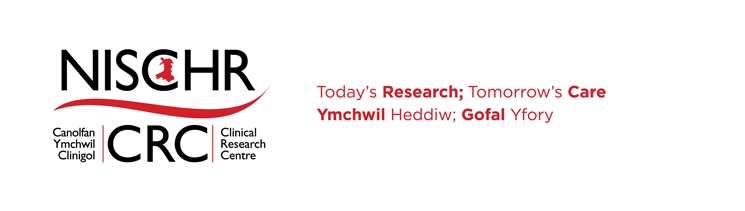

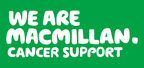


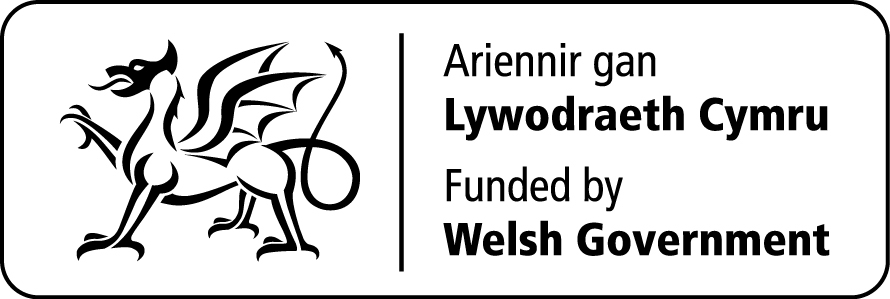


**Building Research Partnerships:**

**Getting Involved and Influencing Research**

18^th^ June 2014

# Evaluation Form

**1. Overall Evaluation**

|  |  | **Poor** | **Satisfactory** | **Good** | **Excellent** | **Any comments** |
| --- | --- | --- | --- | --- | --- | --- |
|  | How would you rate the day overall? |  |  |  |  |  |
|  | How would you rate the programme? |  |  |  |  |  |
|  | How would you rate the organisation of the day? |  |  |  |  |  |
|  | How would you rate the day in terms of relevance to your work? |  |  |  |  |  |
|  | How would you rate the influence of the day on your research practice/or involvement in research? |  |  |  |  |  |

Please give examples of how this training day will influence your research practice or involvement in

research**:**

1.______________________________________________________________________________________

2.______________________________________________________________________________________

3.______________________________________________________________________________________

**Please turn over**

**2. Individual Sessions**

**Please rate the quality of each session.**

|  | **Sessions** | **Poor** | **Satisfactory** | **Good** | **Excellent** | **Comments** |
| --- | --- | --- | --- | --- | --- | --- |
|  | What is research? |  |  |  |  |  |
|  | Research methodology and terminology |  |  |  |  |  |
|  | The research cycle and lay involvement |  |  |  |  |  |
|  | Examples of what is currently being researched |  |  |  |  |  |
|  | The 6 R’s |  |  |  |  |  |
|  | Personal boundaries |  |  |  |  |  |
|  | Revisit research cycle – Proposals & patient info sheets |  |  |  |  |  |

**Any further comments:**

………………………………………………………………………………………………………………………………

………………………………………………………………………………………………………………………………

**Please identify any further research training you require:**

………………………………………………………………………………………………………………………………

**Job Title:** …………………………………………………………………………………………………………………

**Please return to the facilitator or send to the NISCHR CRC Central Office, 3^rd^ Floor,**

**12 Cathedral Road, Cardiff, CF11 9LJ**

**Fax number 029 2019 6817**

Thank you for completing this evaluation

Mae NISCHR CRC yn rhan o’r isadeiledd ymchwil i Gymru sy’n cael ei ariannu gan NISCHR, Llywodraeth Cymru [www.wales.gov.uk/nischr](http://www.wales.gov.uk/nischr)

NISCHR CRC is part of the research infrastructure for Wales funded by NISCHR, Welsh Government [www.wales.gov.uk/nischr](blocked::http://www.wales.gov.uk/nischr)
