# Supplementary material for: “Hitting the spot”: Developing individuals with lived-experience of health and social care as facilitators to deliver a course to enhance public involvement in research – a Welsh perspective
Source: Res Involv Engagem. 2017 Apr 4;3:5. doi: 10.1186/s40900-017-0057-z (PMC5611615; doi:10.1186/s40900-017-0057-z)
Supplement: Supplementary file 2 — Model of facilitator development (DOCX 13 kb) [file 40900_2017_57_MOESM2_ESM.docx]

**Model of facilitator development**

- **Memorandum of Understanding developed between Macmillan Cancer Support and Health and Care Research Wales (who does what and how costs/ expenses are met)**
- **Trainee facilitators attend as delegates at a course delivered by an experienced facilitator**
- **Pre-course meeting with mentor (experienced facilitator), including going through the facilitator manual**
- **First course delivered, led by mentor with contributions from trainee facilitators**
- **Post-course feedback, evaluation and review**
- **Facilitator training day provided (for all new facilitators on the Health and Care Research Wales training programme)**
- **Second course delivered, led by mentor with contributions from 2 trainee facilitators (plus pre-/post- course meetings)**
- **Third / fourth course delivered – trainee facilitators fully co-facilitating and leading specific sessions (pre-/post-review)**
- **One-to-one meetings between facilitators and project lead to get feedback and assess any further training needs (interim report to Macmillan)**
- **Project review meeting with all three facilitators, Macmillan and Health and Care Research Wales**
- **Fifth course delivered – trainee facilitators leading the whole day (mentor observing)**
- **Final report to Macmillan**
- **Completion of facilitator training and certification**
- **Continuing course delivery without mentor (co-facilitation approach).**
