# Supplementary material for: “Hitting the spot”: Developing individuals with lived-experience of health and social care as facilitators to deliver a course to enhance public involvement in research – a Welsh perspective
Source: Res Involv Engagem. 2017 Apr 4;3:5. doi: 10.1186/s40900-017-0057-z (PMC5611615; doi:10.1186/s40900-017-0057-z)
Supplement: Supplementary file 3 — Self-evaluation form (DOCX 14 kb) [file 40900_2017_57_MOESM3_ESM.docx]

**Self-evaluation form**

**Why are we collecting this information?** We've designed this form to be a useful tool to gather feedback from trainers and facilitators about courses and workshops they are running. This will help us improve our learning events and the support we can offer trainers and facilitators. It is also hopefully a useful chance to reflect on any training that you have done and provide a personal record.

**What will we do with this information?** All information you provide will be treated in confidence and held in accordance with the Data Protection Act 1998. It will not be shared with participants and other trainers and facilitators but may be shared between Macmillan staff and relevant staff at partner organisations when appropriate. When possible, any information shared.

**How to use this form**: Please fill it in and save it with your name and date in the file name. The space provided is not a guide, so please write as much as you feel appropriate. If you would like this document in other formats (e.g. paper/online) please contact your main contact at the centre where you delivered the event.

1. What is your name?
2. What is the name of the person who asked you to facilitate this event?
3. What was the name of the course or event?
4. What was the date and location of this event?
5. Overall success: do you think the learning objectives were achieved?
6. Engagement of the group in activities: were the activities understood, relevant, lively and challenging enough?
7. Engagement of the group in discussions: did everyone participate and did they listen to each other?
8. Smooth running: did you have everything you needed to deliver the training? Would you have liked any support or additional materials?
9. Quality of the materials: how good were they in endorsing the messages you were trying to get across?

Is there anything you would like Macmillan or the centre to do differently that would help you to improve your delivery of training?

1. Were there any particular challenges for you in running this particular workshop with this particular group?
2. Please add any other comments here:
